# New Antibacterial Silver(I) Coordination Polymers Based on a Flexible Ditopic Pyrazolyl-Type Ligand

**DOI:** 10.3390/polym11101686

**Published:** 2019-10-15

**Authors:** Aurel Tăbăcaru, Claudio Pettinari, Mariana Bușilă, Rodica Mihaela Dinică

**Affiliations:** 1 Faculty of Sciences and Environment, Department of Chemistry, Physics and Environment, “Dunarea de Jos” University of Galati, 111 Domneasca Street, 800201 Galați, Romania; 2 School of Pharmacy, University of Camerino, Via Madonna delle Carceri 9, 62032 Camerino MC, Italy; claudio.pettinari@unicam.it; 3 Department of Materials Science and Engineering, Faculty of Engineering, ”Dunarea de Jos” University of Galati, 111 Domneasca Street, 800201 Galați, Romania; mariana.ibanescu@ugal.ro

**Keywords:** silver, coordination polymers, nitrogen ligands, thermal stability, antibacterial activity

## Abstract

In the last two decades, a tremendous amount of attention has been directed towards the design of antibacterial silver(I)-based materials, including coordination polymers (CPs) built up with a great variety of oxygen and nitrogen-containing ligands. Herein, a family of six new silver(I)-based CPs, having the general stoechiometric formula [Ag(H_2_DMPMB)(X)] (X = NO_3_, **1**; CF_3_CO_2_, **2**; CF_3_SO_3_, **3**; BF_4_, **4**; ClO_4_, **5**; and PF_6_, **6**) and incorporating the flexible ditopic pyrazolyl-type ligand 4,4′-bis((3,5-dimethyl-1H-pyrazol-4-yl)methyl)biphenyl (H_2_DMPMB), has been prepared by the chemical precipitation method involving the reaction of silver(I) salts with H_2_DMPMB in the 1:1 molar ratio, in alcohols, or acetonitrile at room temperature for two-hours. The new silver(I)-based polymeric materials were characterized by means of Fourier transform infrared spectroscopy (FTIR), elemental analysis (EA), and thermogravimetric analysis (TGA), allowing for the proposition that their structures comprise one-dimensional chains, with the silver(I) ions mostly assuming a T-shapped stereochemistry completed by the exo-bidentate ligands and counter-anions. The obtained silver(I) CPs showed a remarkable light insensitivity and stability in the air, are insoluble in water and in most common organic solvents, and possess appreciable thermal stabilities spanning the range 250–350 °C. The antibacterial activity of the obtained silver(I) CPs was tested against the Gram-negative bacteria *Escherichia coli* (*E. coli*) and Gram-positive bacteria *Staphylococcus aureus* (*S. aureus*) using the Tetrazolium/Formazan test (TTC), by measuring the bacterial viability at different time intervals. The complete reduction of both bacterial strains occurred after 24 h of exposure to all silver(I) CPs, the bacterial viability values for *S. aureus* reaching 8% for compounds **3**, **5**, and **6** after only two-hours.

## 1. Introduction

Coordination polymers (CPs) [1,2,3,4], and the subset of metal-organic frameworks (MOFs) [5,6,7], represent the vast class of inorganic−organic hybrid materials, which are made of metal ions or metal-based clusters and polydentate ligands connected through coordination bonds, which develop into 1-D, 2-D, and 3-D infinite structures. CPs and MOFs have gained a special place in fields of industrial, technological, economical, and environmental interest thanks to the great variety of functional properties so far achieved, ranging from gas storage and separation [8] to heterogeneous catalysis [9], luminescence [10], sensing [11], magnetism [12], conductivity [13], and biomedicine [14]. For their successful construction, the most typically used organic ligands have been rationally chosen from the class of poly(carboxylates) [15,16], poly(azolates) [17,18,19], pyrazines and bipyridines [15,16,20], and phosphonates [21].

Special attention has also been offered, over the past two decades, to the synthesis of silver(I)-based CPs, which were found to show fascinating structural motifs [22,23,24,25] and interesting properties, ranging from photoluminescence [26,27] to electrical conductivity [28,29], magnetism [30,31], guest exchange and sorption [32,33], and catalysis [34,35]. The successful preparation of such polymeric materials has taken the judicious management of various factors into consideration, such as the reaction conditions [36], the silver-to-ligand ratio [37], the stereochemistry of silver ion coupled with the ligands functionality [38], and the nature of counter-anions [39].

In the last decade, silver(I)-based CPs, built up either with rigid or flexible nitrogen and oxygen-donor ligands, have gained popularity due to their potential as antibacterial agents, which unlike the soluble silver(I) complexes [40], have arisen to minimize the problem of reducing, as much as possible, the amount of silver(I) ions released into the environment, thus relying on their higher stabilities and very low solubilities. The pioneering works by Nomiya et al. have reported positive results of antimicrobial and antifungal activities of the azolyl-based silver(I) CPs [Ag(im)] [41], [Ag(1,2,3-tz)(PPh_3_)_2_] and [Ag(1,2,4-tz)(PPh_3_)_2_] [42], [Ag(tetz)(PPh_3_)_2_] [43] (im = 1,3-imidazolate, 1,2,3-tz = 1,2,3-triazolate, 1,2,4-tz = 1,2,4-triazolate, tetz = 1,2,3,4-tetrazolate, PPh_3_ = triphenylphosphine). These first results have then stimulated the preparation and characterization of new, other types of silver(I) CPs with promising results regarding the antibacterial activity against a wide spectrum of Gram-negative and Gram-positive pathogenic microorganisms. In this context, various prototypes of silver(I) CPs have been tested in the form of powders [44,45,46,47,48,49], polymer-based composites [50,51,52,53,54], and surface coatings [55,56,57,58,59], exhibiting remarkable biocidal effects, either in suspension or by contact.

**Scheme 1:**
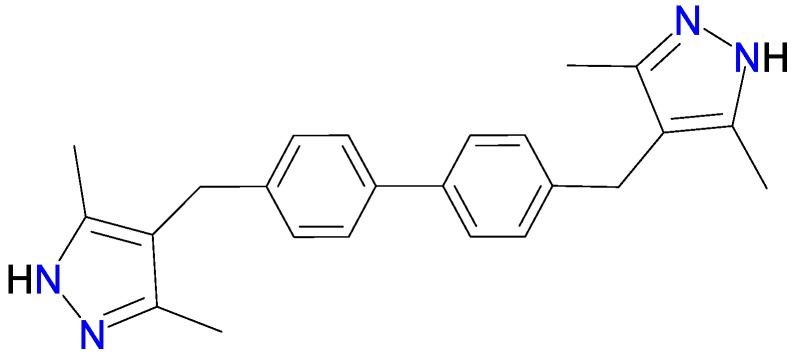
Structure of 4,4′-bis((3,5-dimethyl-1H-pyrazol-4-yl)methyl)biphenyl (H_2_DMPMB).

In the past few years, we have shown that the flexible ditopic pyrazolyl-type ligand 4,4′-bis((3,5-dimethyl-1H-pyrazol-4-yl)methyl)biphenyl (H_2_DMPMB, Scheme 1) [60], and its shorter analogue 1,4-bis((3,5-dimethyl-1H-pyrazol-4-yl)methyl)benzene (H_2_BDMPX) [61], were able to generate cobalt(II)-, zinc(II)-, cadmium(II)-, and copper(I)-containing CPs displaying high thermal stabilities, coupled with either pro-porous properties or photoluminescence. Herein, we sought to further explore the coordinative potentiality of the flexible ligand H_2_DMPMB towards the preparation of new antibacterial silver(I)-based CPs with different counter-anions, which are remarkably light-insensitive and highly insoluble.

The present paper is, therefore, aimed at synthesizing, through a simple chemical precipitation and high-yielding method, a family of six new silver(I)-based CPs incorporating the flexible ligand H_2_DMPMB with the general stoechiometric formula [Ag(H_2_DMPMB)(X)] (X = NO_3_, **1**; CF_3_CO_2_, **2**; CF_3_SO_3_, **3**; BF_4_, **4**; ClO_4_, **5**; and PF_6_, **6**). Only two examples of silver nitrate and perchlorate complexes with the ethyl homologues of the flexible H_2_DMPMB ligand were reported a few years ago [62]. Recently, two photoluminescent silver nitrate 1D coordination polymers with the bitopic ligand 1,1,2,2-tetra(pyrazol-1-yl)ethane were structurally characterized [63]. While Fourier transform infrared spectra (FTIR) were exhaustively analyzed to propose the structure of all the obtained silver(I) polymeric compounds, thermogravimetric analysis (TGA) revealed their appreciable thermal stabilities, lying in the range 250–350 °C. The antibacterial activity of the obtained silver(I) CPs against the Gram-negative bacteria *Escherichia coli* (*E. coli*) and Gram-positive bacteria *Staphylococcus aureus* (*S. aureus*) was tested using the Tetrazolium/Formazan test (TTC), by measuring the bacterial viability at different time intervals.

## 2. Materials and Methods

### 2.1. General

- All reagents and solvents were purchased from Sigma-Aldrich (Darmstadt, Germany) and Alfa Aesar (Kandel, Germany), and were used as received.
- The ligand 4,4′-bis((3,5-dimethyl-1H-pyrazol-4-yl)methyl)biphenyl (H_2_DMPMB) was synthesized according to an optimized method previously reported in the literature [60].
- FTIR spectra were recorded from 4000 to 650 cm^‒1^ with a Perkin–Elmer Spectrum 100 instrument (Perkin-Elmer, Shelton, CT, USA) by total reflectance on a ZnSe crystal. In the following, the IR bands are classified as very weak (vw), weak (w), medium (m), strong (s), and very strong (vs).
- Elemental analyses (C, H, N, and S) were performed in-house with Fisons Instruments 1108 CHNS-O Elemental Analyzer (Thermo Scientific, Waltham, MA, USA). Before performing the analytical characterization, all samples were dried in vacuo (50 °C, ~10^‒4^ bar) until a constant weight was reached.
- Termogravimetric analyses (TGA) were carried out from 30 to 700 °C in a N_2_ stream with a Perkin–Elmer STA 6000 simultaneous thermal analyzer (Perkin-Elmer, Shelton, CT, USA) with the heating rate of 7 °C/min.

### 2.2. Syntheses

[Ag(H_2_DMPMB)(NO_3_)] (1). H_2_DMPMB (0.093 g, 0.25 mmol) was dissolved in 30 mL of methanol under gentle warming (45 °C). Then, AgNO_3_ (0.085 g, 0.5 mmol) was added. The resulting white suspension was left under stirring at room temperature for 2 h, obtaining a white precipitate that was filtered off, washed twice with methanol, and dried under a vacuum. Yield: 75%. **1** is insoluble in chlorinated solvents, alcohols, acetone, acetonitrile, dimethylformamide, dimethylsulfoxide, and water. IR (cm^−1^): 3199(mbr) ν(N–H); 3100-3000(vw) ν(C–H_aromatic_), 3000–2900(w) ν(CH_3_, CH_2_), 1748(w), 1582(w), 1528(w), 1499(w) ν(C=C+C=N), 1387(vs) ν_asym_(NO_3_), 1347(vs) ν_asym_(NO_3_), 1210(w), 1006(w), 905(w), and 754(vs). Elemental analysis calcd. for C_24_H_26_AgN_5_O_3_ (FW = 540.36 g/mol): C, 53.34; H, 4.85; N, 12.96%. Found: C, 53.03; H, 4.67; N, 12.38%.

[Ag(H_2_DMPMB)(CF_3_CO_2_)] (2). H_2_DMPMB (0.093 g, 0.25 mmol) was dissolved in 30 mL of ethanol under gentle warming (45 °C). Then, AgCF_3_CO_2_ (0.111 g, 0.5 mmol) was added. The resulting white suspension was left under stirring at room temperature for 4 h, obtaining a white precipitate that was filtered off, washed twice with ethanol, and dried under a vacuum. Yield: 75%. **2** is insoluble in chlorinated solvents, alcohols, acetone, acetonitrile, dimethylformamide, dimethylsulfoxide, and water. IR (cm^−1^): 3186(mbr), ν(N–H), 3092(vw), 3028(vw) ν(C–H_aromatic_), 2921(vw) ν(CH_3_, CH_2_), 1668(vs) ν(C=O), 1579(vw), 1528(vw), 1494(m) ν(C=C+C=N), 1431(m), 1201-1140(vs) ν(CF_3_CO_2_), 1058(m), 1005(m), 905(m), 806(m), 719(s). Elemental analysis calc. for C_26_H_26_AgF_3_N_4_O_2_ (FW = 591.38 g/mol): C, 52.81; H, 4.43; and N, 9.47%. Found: C, 52.35; H, 4.25; N, 9.08%.

[Ag(H_2_DMPMB)(CF_3_SO_3_)] (3). H_2_DMPMB (0.093 g, 0.25 mmol) was dissolved in 30 mL of ethanol under gentle warming (45 °C). Then, AgCF_3_SO_3_ (0.129 g, 0.5 mmol) was added. The resulting white suspension was left under stirring at room temperature for 4 h, obtaining a white precipitate that was filtered off, washed twice with ethanol, and dried under a vacuum. Yield: 70%. **3** is insoluble in chlorinated solvents, alcohols, acetone, acetonitrile, dimethylformamide, dimethylsulfoxide, and water. IR (cm^−1^): 3233(mbr), ν(N–H), 3024(vw) ν(C–H_aromatic_), 2923(vw) ν(CH_3_, CH_2_), 1586(w), 1526(vw), 1498(w) ν(C=C+C=N), 1240(vs), 1224(vs), 1162(vs), 1029(vs) ν(CF_3_SO_3_), 905(w), 839(w), and 759(m). Elemental analysis calcd. for C_25_H_26_AgF_3_N_4_O_3_S (FW = 627.43 g/mol): C, 47.86; H, 4.18; N, 8.93; S, 5.11%. Found: C, 47.85; H, 3.92; N, 8.34; S, 4.91%.

[Ag(H_2_DMPMB)(BF_4_)] (4). H_2_DMPMB (0.093 g, 0.25 mmol) was dissolved in 30 mL of acetonitrile under gentle warming (45 °C). Then, (MeCN)_4_AgBF_4_ (0.179 g, 0.5 mmol) was added. The resulting white suspension was left under stirring at room temperature for 4 h, obtaining a white precipitate that was filtered off, washed twice with acetonitrile, and dried under a vacuum. Yield: 80%. **4** is insoluble in chlorinated solvents, alcohols, acetone, acetonitrile, dimethylformamide, dimethylsulfoxide, and water. IR (cm^−1^): 3316(mbr) ν(N–H), 3025(vw) ν(C–H_aromatic_), 2923(vw) ν(CH_3_, CH_2_), 1587(w), 1526(vw), 1494(w) ν(C=C+C=N), 1057(vs), 1005(vs) ν(BF_4_), 904(w), 845(m), 799(w), 768(m), and 692(mbr). Elemental analysis calcd. for C_24_H_26_AgBF_4_N_4_ (FW = 565.17 g/mol): C, 51.00; H, 4.64; N, 9.91%. Found: C, 50.86; H, 4.45; N, 9.36%.

[Ag(H_2_DMPMB)(ClO_4_)] (5). H_2_DMPMB (0.093 g, 0.25 mmol) was dissolved in 30 mL of ethanol under gently warming (45 °C). Then, AgClO_4_ (0.104 g, 0.5 mmol) was added. The resulting white suspension was left under stirring at room temperature for 2 h, obtaining a white precipitate that was filtered off, washed twice with ethanol, and dried under a vacuum. Yield: 85%. **5** is insoluble in chlorinated solvents, alcohols, acetone, acetonitrile, dimethylformamide, dimethylsulfoxide, and water. IR (cm^−1^): 3267(mbr) ν(N–H), 3025(vw) ν(C–H_aromatic_), 2923(vw) ν(CH_3_, CH_2_), 1587(w), 1525(vw), 1495(w) ν(C=C+C=N), 1075(vs), 1040(vs) ν(ClO_4_), 845(m), 769(m), and 687(mbr). Elemental analysis calcd. for C_24_H_26_AgClN_4_O_4_ (FW = 577.82 g/mol): C, 49.89; H, 4.54; N, 9.70%. Found: C, 49.52; H, 4.46; N, 9.51%.

[Ag(H_2_DMPMB)(PF_6_)] (6). H_2_DMPMB (0.093 g, 0.25 mmol) was dissolved in 30 mL of ethanol under gently warming (45 °C). Then, AgPF_6_ (0.125 g, 0.5 mmol) was added. The resulting white suspension was left under stirring at room temperature for 3 h, obtaining a white precipitate that was filtered off, washed twice with ethanol, and dried under a vacuum. Yield: 90%. **6** is insoluble in chlorinated solvents, alcohols, acetone, acetonitrile, dimethylformamide, dimethylsulfoxide, and water. IR (cm^−1^): 3410(mbr) ν(N-H), 3027(vw) ν(C–H_aromatic_), 2926(vw) ν(CH_3_, CH_2_), 1585(w), 1525(vw), 1495(w) ν(C=C+C=N), 828(vs) ν(PF_6_), 768(m), and 667(mbr). Elemental analysis calcd. for C_24_H_26_AgF_6_N_4_P (FW = 623.33 g/mol): C, 46.25; H, 4.20; N, 8.99%. Found: C, 46.22; H, 3.98; N, 8.50%.

### 2.3. Bacteria Strain and Growth Conditions

The pathogenic bacteria, *Escherichia coli* ATCC 25922 and *Staphylococcus aureus* ATCC 25923 (clinical isolates), used as test microorganisms for the antibacterial activity study, were collected from the Microbial Culture Collection (Sf. Andrei Hospital of Galati, Romania). The cultivation medium for *E. coli* and *S. aureus* was Brain Heart Infusion Broth (BHI, Merck, Darmstadt, Germany). Bacterial culture for antibacterial activity was prepared by picking the colony in BHI agar plates and incubated for 24 h, followed by suspension in an appropriate medium (9 mL). The culture was grown aerobically for 20 h at 37 °C. For antibacterial assay, 1 mL of bacterial culture was diluted in 9 mL BHI medium to 10^6^ CFU/mL.

### 2.4. Antibacterial Activity

TTC (2,3,5-triphenyl tetrazolium chloride, Merck) was prepared by dissolving the powder in sterile water (5 mg/mL) at room temperature. In the presence of bacteria, TTC was reduced to red formazan, which was directly proportional to the activity and viability of the bacterial cells [64]. The TTC test was evaluated as a qualitative antibacterial method. For the susceptibility test using TTC, 1 mL of silver(I) CPs (suspended in sterile water, 5 mg/mL), 1 mL of AgNO_3_ used as positive control (dissolved in sterile water, 5 mg/mL), and 100 µL of bacteria suspension (10^6^ CFU/mL) was added into 40 mL of BHI broth. The control sample contained only bacterial suspension and BHI broth. All flasks were incubated with shaking at 37 °C at 200× *g* for 4 h. Then, 1 mL from each flask containing the treated and control cultures was transferred to sterile Eppendorf tubes, and 100 µL of TTC was then introduced. All tubes were incubated at 37 °C for 20 min. The samples were centrifuged at 4000× *g* for 3 min, followed by decantation of the supernatants. The resulting formazan crystals were resuspended in ethanol (70%) and centrifuged again. The red formazan solution obtained at the end was measured by a microplate reader (Tecan Infinite 200PRO, Tecan Trading AG, Männendorf, Switzerland) at 480 nm. All experiments were done in triplicate and the relative cell viability (%) was represented by a percentage relative to the untreated control cells.

## 3. Results and Discussion

### 3.1. Synthesis and FTIR Spectroscopy

Compounds **1–6**, having the general stoechiometric formula [Ag(H_2_DMPMB)(X)] (X = NO_3_, **1**; CF_3_CO_2_, **2**; CF_3_SO_3_, **3**; BF_4_, **4**; ClO_4_, **5**; and PF_6_, **6**), hypothesized with the help of elemental analyses, have been obtained from the room temperature reaction carried out in a 2:1 molar ratio of two equivalents of silver(I) salts and one equivalent of H_2_DMPMB, in alcohol for compounds **1–3**, **5,** and **6**, and acetonitrile for compound **4**. Their stoechiometry was found to be independent of the amount of silver(I) salts or ligand used in the syntheses, since the 1:1 molar ratio was always obtained, which was also observed in other bis(pyrazolyl)-based silver(I) compounds, such as [Ag(H_2_BPZ)(X)] (X = NO_3_, ClO_4_, BF_4_, PF_6_, CH_3_SO_3_, CF_3_SO_3_; H_2_BPZ = 4,4′-bipyrazole) [54], [Ag(H_2_Me_4_BPZ)(NO_3_)]∙CH_3_OH [65], and [Ag(H_2_Me_4_BPZ)(X)] (X = ClO_4_, CF_3_SO_3_, C_2_F_5_CO_2_; H_2_Me_4_BPZ = 3,3′,5,5′-tetramethyl-4,4′-bipyrazole) [66]. 

All compounds were in the form of white powders, and were remarkably light insensitive and air stable, even after twelve months, thus relying on the strength of silver-ligand bonds that may be imparted to the inertness of the overall structure. In fact, no change of their aspect was noticed, and both elemental analyses and FTIR spectra were preserved. Their polymeric nature was indicated by the observed insolubility, both in water and in most common organic solvents, along with the occurrence of high thermal decomposition temperatures revealed by the thermogravimetric analysis (Section 3.2). A preliminary powder X-ray diffraction analysis showed the amorphous nature of the obtained silver(I) CPs, and therefore, their molecular structures are yet to be revealed. Nevertheless, a deeper analysis of their FTIR spectra allowed the proposition of a structural characterization for the obtained H_2_DMPMB-based silver(I) CPs, as will be shown in the following lines.

**Figure 1:**
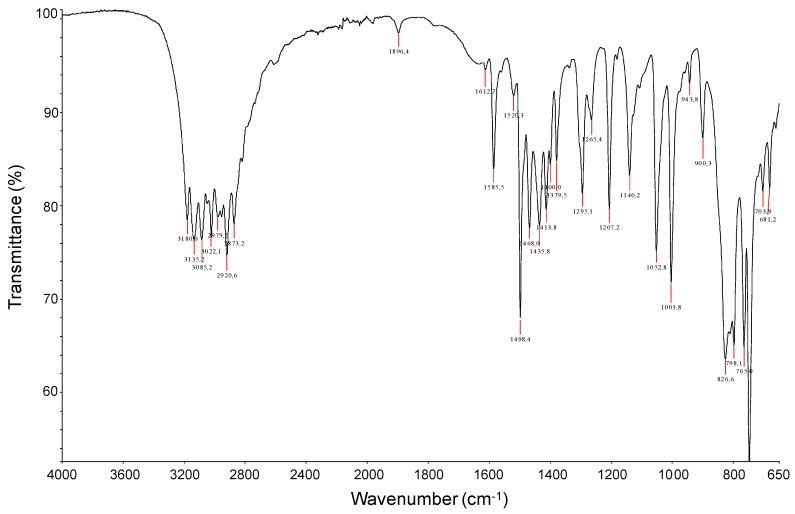
Fourier transform infrared spectroscopy (FTIR) spectrum of the free H_2_DMPMB ligand.

**Figure 2:**
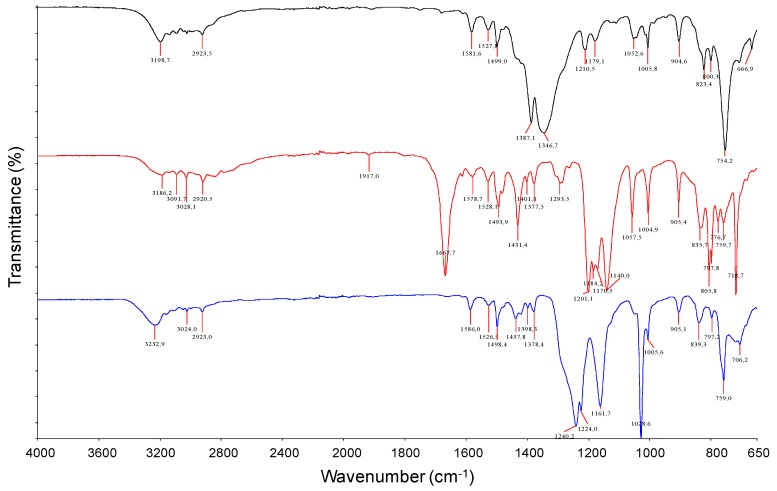
FTIR spectra of silver(I) CPs **1** (black), **2** (red), and **3** (blue).

**Figure 3:**
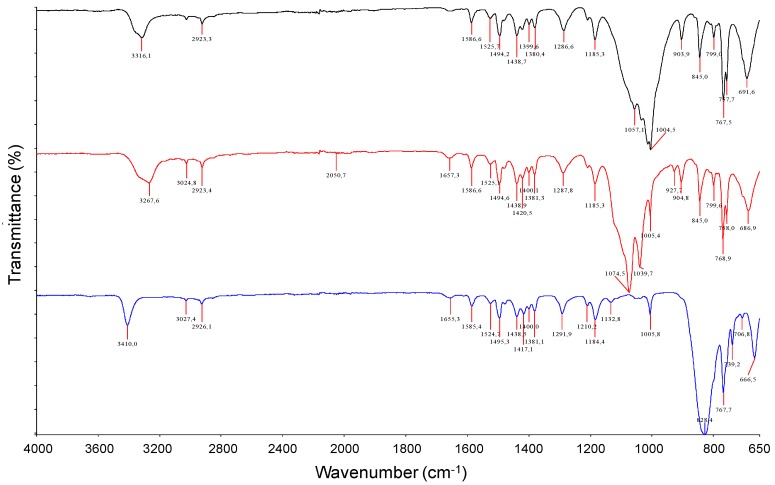
FTIR spectra of silver(I) CPs **4** (black), **5** (red), and **6** (blue).

FTIR spectroscopy was employed in order to reveal both the changes in the absorbtion bands of the ligand upon coordination to silver(I) ions and the binding modes of the counterions. The FTIR spectrum of the free H_2_DMPMB ligand is given in Figure 1, while the FTIR spectra of silver(I) CPs **1–6** are given in Figure 2 and Figure 3. The infrared spectrum of the free ligand showed a strong and broad band in the region 2500–3200 cm^−1^, which was specific to the intermolecular hydrogen bond interactions mediated by the N‒H functions of the pyrazolyl rings. Upon coordination to silver(I) ions, the infrared pattern of the ligand was significantly changed, indicating the success of the synthetic methodology adopted for obtaining the new silver(I) CPs.

The infrared spectra of all silver(I) CPs were generally consistent with the proposed formulations, and present all of the required absorbtion bands for both the ligand and the counterions. The presence of absorption bands, with a generally medium intensity in the range of 3180–3410 cm^−1^, was specific for the stretching vibrations of the N–H function, confirming that the ligand maintained its neutrality in all compounds. The weak absorption bands located in the range 1579–1587 cm^−1^ were due to the so-called “breathing” of the neutral pyrazolyl rings [67], which was significantly reduced compared to the free ligand, as a consequence of coordination, and in consideration of the influence manifested by the different anions.

**Figure 4:**
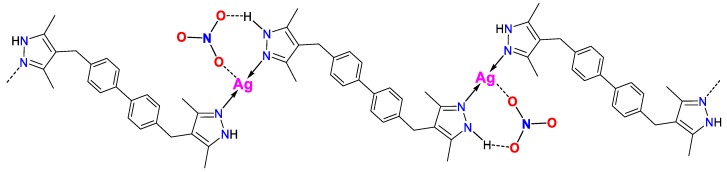
Proposed polymeric structure of [Ag(H_2_DMPMB)(NO_3_)] (**1**), evidencing the T-shaped stereochemistry of silver(I) ions assured by the nitrate anions and the neutral ligands coordinated in a bridging exo-bidentate mode.

A deeper analysis of the infrared spectra of all synthesized silver(I) CPs was also performed in order to obtain clues about the possible interactions in which the anions are involved within the corresponding structures. As such, in the IR spectrum of **1,** the asymmetric stretching modes of the nitrate anion were denounced by the slightly split bands at 1387 and 1347 cm^−1^. Moreover, the single, very weak band, almost indistinguishable from noise, in the overtone region at 1748 cm^−1^**,** suggested that the nitrate anion was in a non-coordinated form [68]. Such a suggestion was further strengthened by the absence of the band associated with symmetric stretching nitrate vibrations near 1049 cm^−1^ [69]. The broad band present in the region 2700–3400 cm^−1^ was indicative of the hydrogen bond interactions between the nitrate anion and the N–H function of the ligand, spreading along adjacent linear chains formed by the [Ag(H_2_DMPMB)]^+^ units. However, although the nitrate anion was not directly bound to the silver(I) ion, weak Ag‒O interactions could be considered so that the silver(I) ion assumes a T-shaped stereochemistry (Figure 4), as was already observed in the silver(I) CPs [Ag(H_2_BPZ)(NO_3_)] [54] and [Ag(Me_4_BPZ)(NO_3_)]∙CH_3_OH [65], and in the silver(I) complexes AgNO_3_:tz_2_(CH_2_):ER_3_:MeCN (tz_2_(CH_2_) = bis(1,2,4-triazol-1-yl)methane; E = P, As, Sb; MeCN = acetonitrile) [70].

**Figure 5:**
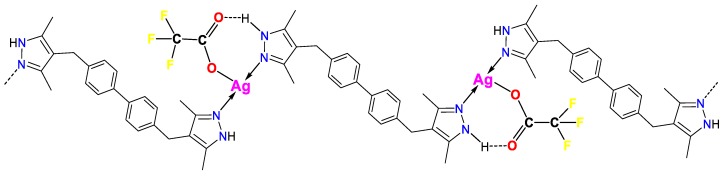
Proposed polymeric structure of [Ag(H_2_DMPMB)(CF_3_CO_2_)] (**2**), evidencing the T-shaped stereochemistry of silver(I) ions assured by the trifluoroacetate anions and the neutral ligands coordinated in a bridging exo-bidentate mode.

**Figure 6:**
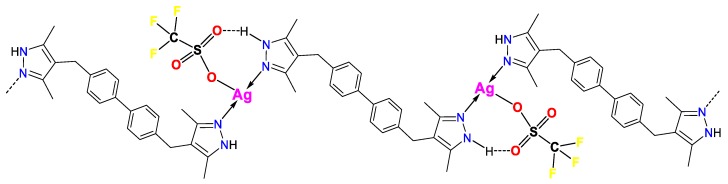
Proposed polymeric structure of [Ag(H_2_DMPMB)(CF_3_SO_3_)] (**3**), evidencing the T-shaped stereochemistry of silver(I) ions assured by the trifluoromethanesulfonate anions and the neutral ligands coordinated in a bridging exo-bidentate mode.

The IR spectra of **2** showed a strong band at 1668 cm^−1^ and a medium one at 1431 cm^−1^ that were characteristic of the carboxylate asymmetric (ν_a_) and symmetric stretching (ν_s_), respectively. The difference Δν = ν_a_ – ν_s_ = 237 cm^−1^ likely indicates a unidentate coordination of trifluoroacetate anion [71], thus conferring to the silver(I) ions a T-shaped stereochemistry, as was also observed in the silver(I) CP [Ag(Me_4_BPZ)(C_2_F_5_CO_2_)] [66] and the binaphthylbis(amidopyridyl)-based silver(I) CP [Ag(C_20_H_12_{NHC(O)-3-C_5_H_4_N}_2_)(CF_3_CO_2_)] [72]. The broad band located in the region 2700–3400 cm^−1^ was also found in the IR spectrum of **2**, which was indicative of the hydrogen bond interactions between the trifluoroacetate anion and the N–H function of the ligand (Figure 5). Strong absorbtion bands present in the range 1140–1201 cm^−1^ were assignable to different vibration modes of the C‒F bonds, while the sharp band at 719 cm^−1^ could be attributed to the C‒O vibration [73]. The IR spectra of **3** shows two very strong and broad absorption bands in the 1200–1300 cm^−1^ region, due to asymmetric and symmetric vibrations of the SO_3_ group from the unidentate trifluoromethanesulfonate group, along with the strong and broad absorption at 1162 cm^−1^ due to the C‒F vibrations, and a sharp absorption at 1029 cm^−1^, which was assignable to the S‒O vibration [74,75]. The structure of **3** would therefore be made up of linear chains formed by [Ag(H_2_DMPMB)]^+^ units, in which the silver(I) ions assume a T-shaped stereochemistry assured by the exo-bidentate ligands and the trifluoromethanesulfonate anions, as was also observed in the silver(I) CPs [Ag(H_2_BPZ)(CF_3_SO_3_)] [54] and [Ag(Me_4_BPZ)(CF_3_SO_3_)] [66] (Figure 6).

**Figure 7:**
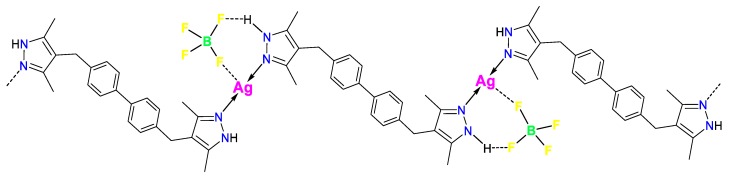
Proposed polymeric structure of [Ag(H_2_DMPMB)(BF_4_)] (**4**), evidencing the T-shaped stereochemistry of silver(I) ions assured by the tetrafluoroborate anions and the neutral ligands coordinated in a bridging exo-bidentate mode.

**Figure 8:**
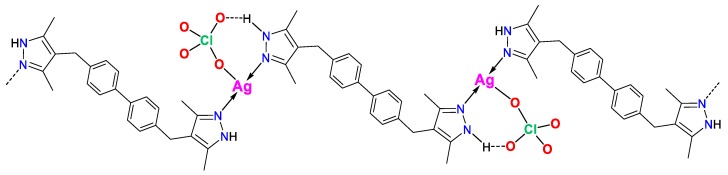
Proposed polymeric structure of [Ag(H_2_DMPMB)(ClO_4_)] (**5**), evidencing the T-shaped stereochemistry of silver(I) ions assured by the perchlorate anions and the neutral ligands coordinated in a bridging exo-bidentate mode.

**Figure 9:**
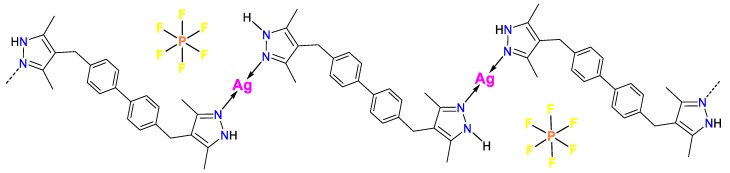
Proposed polymeric structure of [Ag(H_2_DMPMB)(PF_6_)] (**6**), evidencing the digonal stereochemistry of silver(I) ions assured by the neutral ligands coordinated in a bridging exo-bidentate mode.

In the IR spectrum of **4**, the fine splitting of the very strong and broad band in the region 1000–1100 cm^−1^, together with the medium sharp band at 768 cm^−1^ and the medium broad band at 692 cm^−1^, were assigned to the weakly coordinating tetrafluoroborate group (Figure 7) [76], which could be involved in long interactions with the silver(I) ions to complete a T-shaped stereochemistry, as was also observed in the silver(I) CP [Ag(H_2_BPZ)(BF_4_)] [54]. The IR spectrum of **5** showed two strong broad bands at 1040 and 1075 cm^−1^, along with the medium sharp band at 769 cm^−1^ and a medium broad band at 687 cm^−1^, which were attributed to the coordinated perchlorate group (Figure 8) [76], thus completing a T-shaped stereochemistry of the silver(I) ions, as was also observed in the silver(I) CPs [Ag(H_2_BPZ)(ClO_4_)] [54] and [Ag(Me_4_BPZ)(ClO_4_)] [66]. Finally, in the IR spectrum of **6**, the very strong, broad absorption band located at 828 cm^−1^, together with the medium sharp band at 768 cm^−1^ and the medium broad band at 667 cm^−1^ could be assigned to the weakly coordinating hexafluorophosphate anions, which are in fact situated between the cationic sheets instead of being in direct interaction with the silver(I) ions (Figure 9) [77].

### 3.2. Thermogravimetric Analysis

**Figure 10:**
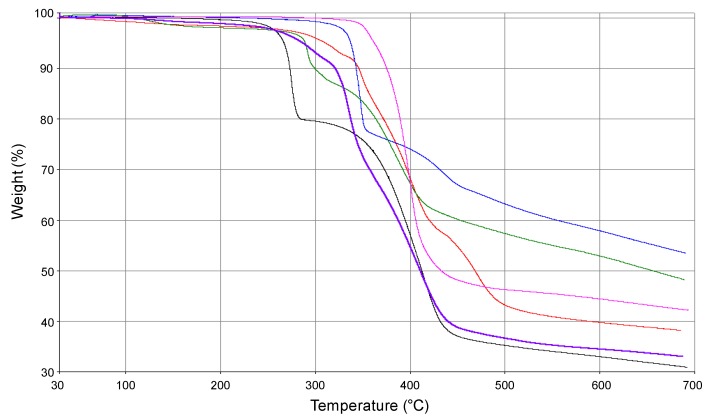
Thermogravimetric analysis (TGA) curves of silver(I) CPs **1** (green), **2** (black), **3** (fuchsia), **4** (red), **5** (blue), and **6** (purple).

The thermogravimetric analyses (TGA) of silver(I) polymeric species **1–6** were performed in order to investigate their thermal behavior upon heating from 30 to 700 °C, under nitrogen, and the resulting TGA curves were collectively gathered in Figure 10. Compound **1** showed an appreciable thermal stability, resistant up to 275 °C, after which a weight loss of about 12% in the range 275–325 °C was recorded, corresponding to the removal of one nitric acid molecule, which was the result of the proton transfer from one pyrazolyl moiety (theoretical weight loss: 11.66%). This loss was immediately followed by a progressive decomposition of the remaining species.

Compound **2** was thermally stable up to 250 °C, which then underwent a weight loss of about 20% until 300 °C, which is in a good agreement with the expected theoretical weight loss of 19.28%, corresponding to the evolution of one trifluoroacetic acid molecule, which was the result of the proton transfer from the organic ligand. This event was further continued by a progressive decomposition of the remaining species. 

Compounds **3** and **5** displayed the highest thermal stabilities, which were resistant up to 350 and 325 °C, respectively, overcoming the values reported for, e.g., the 3-D supramolecular isomer α-[Ag_2_(Me_4_BPZ)] [78] and the neutral CP [Ag_2_(DMPMB)] [60], which were both resistant up to 300 °C, or the 1-D CP [Ag(pz)] (pz = pyrazole), which was stable up to 270 °C [79]. Such remarkable results may be ascribed to the stronger electron-withdrawing effect of both trifluoromethanesulfonate and perchlorate anions, imparting more inertness to the Ag‒N coordinative bonds, and thus to the overall polymeric structures. Compounds **4** and **6** showed the lowest thermal stability, which, although still significant, peaked up to 250 °C, followed then by a progressive decomposition. 

In all cases, at the end of the heating process, black residues, possibly containing a mixture of carbonaceous species and metallic silver/silver(I) oxide, were recovered. Other than the effect of the anions upon the thermal robustness of the obtained silver(I) polymeric species, the dependency of such property on the ligand itself should not be neglected.

### 3.3. Antibacterial Activity

The assessment of antibacterial activity of all prepared silver(I) CPs against Gram-negative bacteria *E. coli* and Gram-positive bacteria *S. aureus* was performed by using the Tetrazolium/Formazan test (TTC) as a qualitative assay, by measuring the bacterial viability at different time intervals (2, 4, 6, and 24 h). The tetrazolium salts are known for their ability to form, within the bacterial culture medium, highly colored products called formazans [80].

From the point of view of the mechanism of antibacterial action, it was shown that, from within the silver-based topical antibacterial agents, the silver(I) ions are those that manifest the bactericidal effects. More exactly, the silver(I) ions prevented the performance of the function of bacterial cells by direct interaction with the bacterial enzymes and DNA, thus interrupting the cell division and replication [81]. On the other hand, the silver(I) ions are involved in the interaction with the negatively charged peptidoglycan from the bacterial cell wall, thus affecting the cell breathing, which finally leads to cell death through the release, into the surrounding environment, their fluid and electrolyte content [82,83]. 

**Figure 11:**
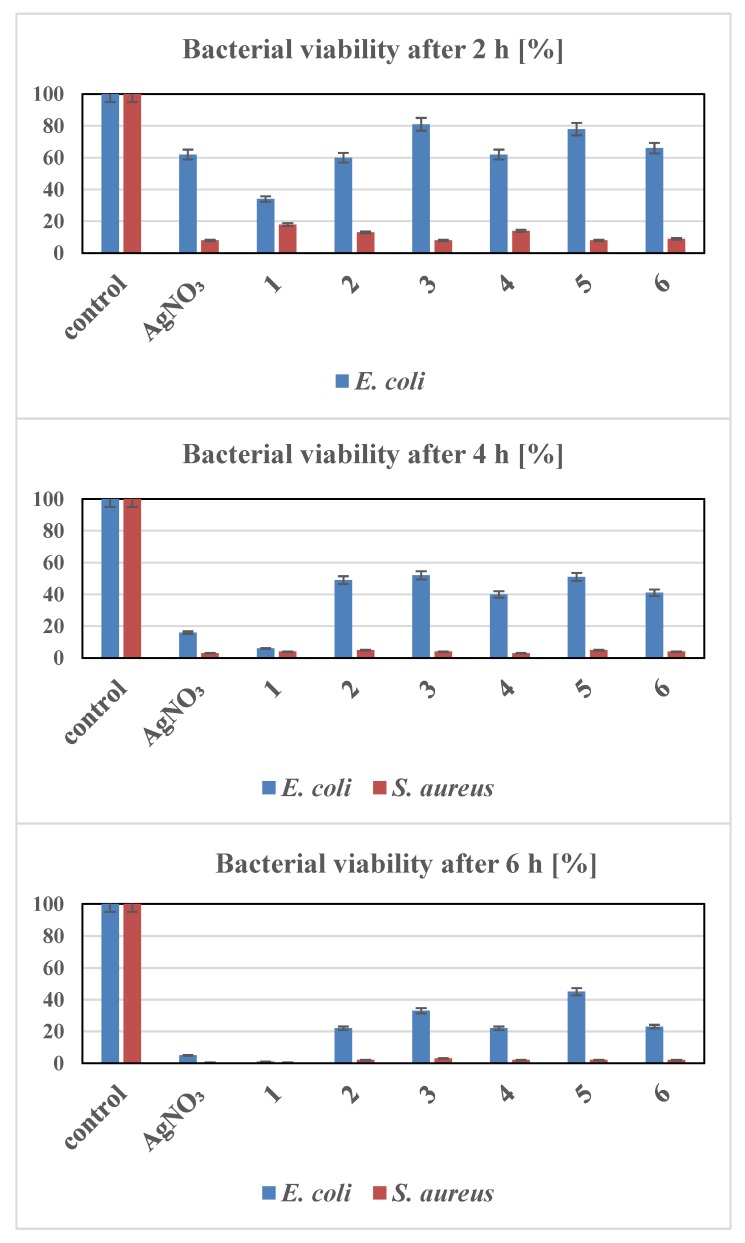
Bacterial viability of *S. aureus* and *E. coli* in the presence of silver(I) CPs **1–6** and standard AgNO_3_.

Figure 11 shows the bacterial viability of the two bacterial strains, *S. aureus* and *E. coli*, in the presence of silver(I) CPs **1–6**, and silver(I) nitrate (AgNO_3_) used as a standard antibacterial agent, after 2, 4, and 6 h. The TTC test indicated, as expected in the presence of an antibacterial agent, the bacterial reduction in time with respect to the control (untreated cells). In the case of *S. aureus*, after 2 h, the values of bacterial viability significantly decreased to 8–19%, and after 6 h the values went down to 1–4%. 

A markedly different behavior was appreciated against *E. coli*, that is, all six silver(I) CPs were less effective in the first period of exposure with a slower action rate compared to *S. aureus*. As such, after 2 h, the values of bacterial viability decreased to 37% (**1**), 60–64% (**2**, **4**, and **6**), and 79–81% (**3** and **5**). The decreasing trend was maintained after 4 h, and after 6 h, the values decreased to 2% (**1**), 21–22% (**2**, **4**, and **6**), 37% (**3**), and 42% (**5**). After 24 h of exposure, a complete bacterial reduction was observed. However, no neat influence of the counter-anions upon the bactericidal effect of the related silver(I) CPs could also be noticed.

For the standard AgNO_3_, the bactericidal effect against *S. aureus* was strong during the first 2 h of exposure (8%), while the bacterial reduction was complete after only 6 h, compared to *E. coli,* where the bacterial reduction was only 62% after 2 h and 5% after 6 h of exposure. Thus, a difference in the rate of action as a function of the bacteria types could be appreciated. It can be observed that the bactericidal effect of the standard AgNO_3_ was comparable with that of our silver(I) CPs. However, the high insolubility of these CPs would bring the advantage of applying them for much longer time intervals, compared to the highly soluble AgNO_3_ (2160 g/L at 20 °C), which underwent faster silver(I) ions release into the environment, thus leading to an increased level of toxicity [52,53,54].

## 4. Conclusions

Coupling the flexible ditopic ligand 4,4′-bis((3,5-dimethyl-1H-pyrazol-4-yl)methyl)biphenyl (H_2_DMPMB) to different silver(I) salts afforded the preparation of six new, light insensitive, air stable, and highly insoluble silver(I) coordination polymers (CPs) with the general formula [Ag(H_2_DMPMB)(X)] (X = NO_3_, **1**; CF_3_CO_2_, **2**; CF_3_SO_3_, **3**; BF_4_, **4**; ClO_4_, **5**; and PF_6_, **6**). The obtained silver(I) CPs demonstrated important antibacterial performances against *E. coli* and *S. aureus*, for which a complete reduction of both bacterial strains occurred after 24 h of exposure to all silver(I) CPs, the bacterial viability value for *S. aureus* reached 8% for compounds **3**, **5,** and **6** after only 2 h. The high insolubility displayed by the new silver(I) CPs presents the advantage, if clinical applications are sought, of reapplying them for much longer time intervals, compared to the highly soluble silver(I) nitrate used as a standard antibacterial agent. The results thus highlight the quality of the new silver(I) CPs as interesting platforms to explore new antibacterial agents. The antibacterial properties proven by our silver(I) CPs, on the inhibition of antibiotic-resistant strains, such as *E. coli* and *S. aureus*, are promising tools that we hope offer a more powerful solution to the worldwide problem that has become a worrying issue: The multiplication of highly multidrug-resistant bacteria in clinical medicine. Future studies will be devoted to the application of these silver(I) CPs into antimicrobial cellulose papers for food packaging protection.
